# Immunotherapies Targeting Tumor-Associated Macrophages (TAMs) in Cancer

**DOI:** 10.3390/pharmaceutics16070865

**Published:** 2024-06-27

**Authors:** Mei-Ye Li, Wei Ye, Ke-Wang Luo

**Affiliations:** 1 School of Pharmaceutical Sciences, Southern Medical University, Guangzhou 510515, China; meiyeli2023@gmail.com (M.-Y.L.); yeyewei2023@gmail.com (W.Y.); 2 People’s Hospital of Longhua, The affiliated hospital of Southern Medical University, Shenzhen 518109, China

**Keywords:** TAMs, polarization, therapies, clinical application

## Abstract

Tumor-associated macrophages (TAMs) are one of the most plentiful immune compositions in the tumor microenvironment, which are further divided into anti-tumor M1 subtype and pro-tumor M2 subtype. Recent findings found that TAMs play a vital function in the regulation and progression of tumorigenesis. Moreover, TAMs promote tumor vascularization, and support the survival of tumor cells, causing an impact on tumor growth and patient prognosis. Numerous studies show that reducing the density of TAMs, or modulating the polarization of TAMs, can inhibit tumor growth, indicating that TAMs are a promising target for tumor immunotherapy. Recently, clinical trials have found that treatments targeting TAMs have achieved encouraging results, and the U.S. Food and Drug Administration has approved a number of drugs for use in cancer treatment. In this review, we summarize the origin, polarization, and function of TAMs, and emphasize the therapeutic strategies targeting TAMs in cancer treatment in clinical studies and scientific research, which demonstrate a broad prospect of TAMs-targeted therapies in tumor immunotherapy.

## 1. Introduction 

Cancer is a significant health problem and a major cause of death in the world. In the process of cancer development, tumor microenvironment (TME) is widely recognized as dynamically regulating cancer progression and influencing therapeutic efficacy, and it is a complex multicellular environment which includes a variety of immune cells, endothelial cells (ECs), cancer-associated fibroblasts (CAFs), pericytes, and other cell types [1]. Among the TME, immune cells, especially macrophages, have recently become an attractive target for cancer therapy, and achieve desirable results in treatment. 

Macrophages indeed play a crucial role in maintaining tissue homeostasis and host defense [2]. Three main functions of these cells are phagocytosis, exogenous antigen presentation, and immunomodulation through the secretion of cytokines and growth factors [3]. Mature macrophages are differentiated from circulating bone marrow-derived monocytes [4]. Within the TME, macrophages are a significant stromal component and are often referred to as TAMs. TAMs can be found in various types of tumor, and are proven to play a complex function in tumor progression and immune response against cancer. In general, TAMs are classified into two types: classical M1 macrophages and alternative M2 macrophages; M1 macrophages are engaged in mechanistic responses, pathogen removal, and anti-tumor immunity. On the contrary, M2 macrophages have a connection with anti-inflammatory responses, wound healing, and the properties of tumorigenicity. TAMs have received widespread attention over the past decades due to their effects on leukocytes, cytokines, and inflammatory mediators. Currently, drugs targeting TAMs include pexidartinib, which produces promising therapeutic outcomes in the treatment of patients with solid tumors. Lee et al. indicated that patients with advanced solid tumors, and treated with 600 mg/day of pexidartinib, experienced an objective remission and prolonged survival, with one third of participants experiencing at least a 30% reduction in the sum diameter of the target lesions, and a 66.7% disease-control rate [5]. Furthermore, the US Food and Drug Administration (FDA) declared their approval of pexidartinib for the treatment of adult patients with giant cell tumor of the tendon sheath on 2 August 2019. Based on the good results demonstrated by TAMs in tumor therapy, we address the origins, polarization, and targeted therapies of TAMs, and also summarize recent advances in immunotherapy for the tumor-targeted treatment of TAMs in recent years. 

## 2. Tumor-Associated Macrophages (TAMs)

TAMs are a specific category of macrophages that exist in the TME. It is believed that TAMs are predominantly derived from circulating monocytes. When stimulated by varying factors, these macrophages show variable phenotypes and functions, which are also known as the polarization of TAMs. The plasticity of TAMs allows them to dynamically respond to changes in the TME and adopt different activation states. In the present section, we will concentrate on the origin and polarization of TAMs.

### 2.1. TAMs’ Origins

It has been proposed for a considerable time that TAMs originated from circulating monocytes, and are attracted to tumors through chemotactic signals emitted by the tumor, such as C-C motif chemokine ligand 2, CCL2) [6]. This single origin of macrophages was not questioned until the discovery of the embryonic origin of tissue-resident macrophages [7]. Increasing evidence suggests that, in addition to circulating monocytes, embryo-derived TAMs are a non-negligible source of TAMs in TME. Although it is suggested that monocyte-derived macrophages could support the growth of TAMs in the inflammatory environment of a tumor, the potentially different contributions of monocyte- and embryo-derived TAMs to tumorigenesis remain a fascinating question.

Circulating monocytes originate from hematopoietic stem cells in bone marrow, such as intestinal macrophages [8]. Yolk sac macrophages and fetal liver could localize to specific tissues and then evolve into functional tissue-resident macrophages [9]. The latest available evidence demonstrates that the recruitment of circulating monocytes is critical for the accumulation of TAMs [10,11,12]. Chemokines, cytokines, and products of the complement cascade [13] are major determinants of macrophage recruitment and positioning in tumors [12]. Also, complement components, especially C5a, are linked to the recruitment and functional polarization of TAMs [14]. The coexistence of circulating monocytes and tissue-resident macrophages has been confirmed in tumor types such as breast tumors [11], brain tumors [15], lung cancer [8], and hepatocellular carcinoma [16]. In lung, brain, and pancreas tumors, TAMs from hematopoietic stem cells express antigen–presenting–related genes, and exhibit extensive immunosuppression, while embryonic–origin–TAMs–expressed genes are closely related to tissue remodeling and wound healing, suggesting that macrophages from different sources produce different physiological effects, even in the same tissue [17,18]. Different sources of TAMs are one of the important causes of the complexity of TME.

### 2.2. TAMs Polarization

TAMs polarization is the process of macrophages exhibiting characteristic phenotypic and functional responses to microenvironmental stimuli and signals encountered by each particular tissue. In this section, we will concentrate on the polarization of TAMs.

**Figure 1:**
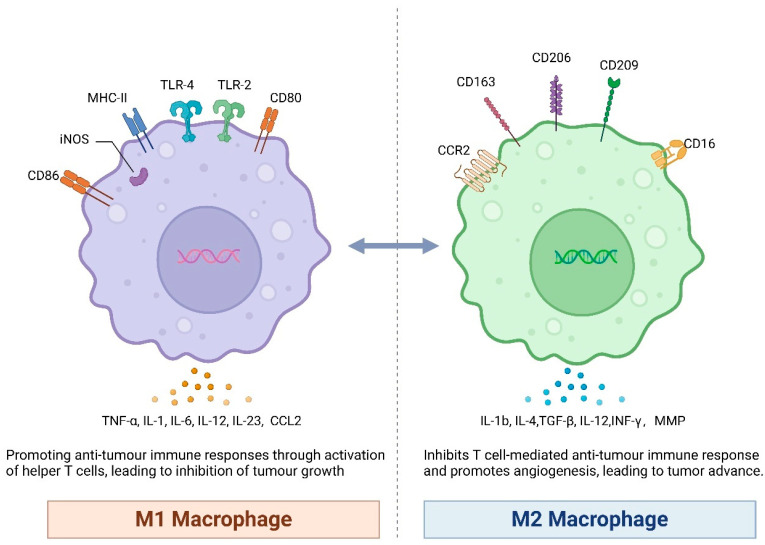
Two major subtypes of TAMs. TAMs are a type of immune cell that infiltrates the tumor microenvironment and plays a significant role in tumor development and progression. TAMs can exhibit different functional phenotypes, often referred to as M1 and M2 polarizations. M1-TAMs are typically associated with an anti-tumor response, and express certain surface markers, such as CD80 and CD86. M1-TAMs produce pro-inflammatory cytokines, such as IL-12, TNF-α. These cytokines contribute to the activation of natural killer cells, cytotoxic T cells, and other immune cells, thereby promoting an anti-tumor immune response. M1-TAMs also produce chemokines like CCL2, which recruit and activate T cells, further enhancing the anti-tumor immune response. On the other hand, M2-TAMs are associated with a pro-tumorigenic function, and express different surface markers, such as CD163 and CD206. M2-TAMs secrete anti-inflammatory cytokines, including TGF-β and IFN-γ, which can suppress immune responses and promote tumor growth. M2-TAMs also produce factors such as MMPs that facilitate tissue remodeling and angiogenesis, supporting tumor invasion and metastasis. It is important to note that the phenotypes and functions of TAMs can be influenced by various factors in the tumor microenvironment, including cytokines, chemokines, and signals from cancer cells themselves. This plasticity allows TAMs to adapt their functions based on the specific conditions within the tumor.

Macrophages are classified into two groups according to their function: classical M1-type macrophages and alternative M2-type macrophages (Figure 1). M1 macrophages are typically activated by lipopolysac charides, viral products, IFN-γ, or granulocyte-macrophage colony-stimulating factor (GM-CSF). M1 produces nitric oxide (NO) and reactive oxygen species (ROS), and also expresses a variety of pro-inflammatory cytokines, for example IL-1, IL-6, IL-12, TNF-α, IL23, and CCL2. M1 also has phagocytosis and could kill target cells. For this reason, M1 macrophages represent a critical cellular component participating in the inflammatory response and anti-tumor immunity. Conversely, M2 macrophages are activated by macrophage colony-stimulating factor (M-CSF), IL-10, IL-4, IL-13, or glucocorticoids [19]. M2 expresses TGF-β, IL-1b, IL-4, IL-12, INF-γ, matrix metalloprotein (MMP), and vascular endothelial growth factor (VEGF) [20], thus it has the ability to repair damaged tissues, stimulate angiogenesis, and promote tumorigenesis and progression [21]. Furthermore, M2 macrophages can be categorized into at least four phenotypes, including M2a, which is induced by IL4 and IL13; M2b, which is induced by immune complexes or LPS; M2c, which is induced by IL10, TGF-β, or glucocorticoids; and M2d, which is the immune-suppressing type of M2 macrophages [22,23].

The main signaling pathways related to M1/M2 polarization are the JAK/STAT signaling pathway, the IRF signaling pathway, the Notch signaling pathway, the PI3K/AKT signaling pathway, and the TLR4 signaling pathway. STAT1 and STAT3/STAT6 are members of the STAT family and play a key role in signal transduction and transcriptional activation. Up-regulation of the suppressor of cytokine signaling (SOCS)1 expression activates the JAK1/STAT1 pathway, which promotes macrophages polarization to the M1 type, leading to an inflammatory response of cytotoxicity and tissue damage [24]. However, activation of STAT3 [25] and STAT6 [26] can polarize macrophages to M2, resulting in immunosuppressive effects. An LPS-induced TLR4 pathway activates JAK2/STAT1, thereby promoting M1 macrophage polarization [27]. M2 TAMs trigger IL-6 production in normal oxytumor cells, which promotes tumorigenesis by regulating JAK-STAT3 [28]. It has been found that the interaction between PPAR γ and the IL-4-STAT6 axis controls the M2 phenotypic transition [29]. In addition, the IRF-signaling pathway has a critical role in the progression of macrophages polarization. Mammals have nine IRFs, and they have significant differences in gene expression and regulation. Related studies have found that IRF-4 specifically regulates M2 cell polarization with the help of histone demethylase Jmjd3 in response to chitin [30]. IRF5 has been shown to be necessary for IL12 and pro-inflammatory cytokine expression [31]. In another study, the expression of IL12 and pro-inflammatory factors in GM-CSF-polarized macrophages was found to be increased along with the up-regulation of IRF5, which in turn activated T cells to stimulate M1 macrophages formation [32]. IRF6 participates in the negative regulation of M2 polarization of mouse bone marrow-derived macrophages by inhibiting PPARγ. In addition, the Notch pathway has an indispensable function in the activation of macrophages polarization. A study found that miR-148a-3p enhanced macrophage M1 polarization and inhibited M2 polarization when activated by Notch [33]. Activation of signal regulatory protein α (SIRPα) can promote macrophage M2 polarization. Notch activation may inhibit the expression of SIRPα through Hes family co-inhibitors, thereby promoting M1 polarization [34]. Apart from that, the PI3/AKT signaling pathway has a critical effect in macrophages polarization. The PI3K/Akt signaling pathway can be affected by TLR4, cytokine chemokines, and the Fc receptor. Activated PI3K I phosphatidylinositol 4,5-bisphosphate (PIP2) produces phosphatidylinositol 3,4,5-triphosphate (PIP3) at the membrane, and the PIP3 further triggers the activation of akt. Activated akt phosphorylates and inactivates the negative regulator of the mechanistic target of rapamycin complex (mTORC)1, thereby activating mTORC1. Consequently, Akt-mTORC1 signaling in macrophages contributes to increased histone acetylation, and generates M2 phenotypes [35]. Ship is a negative modulator of PI3K/Akt signaling; lacking ship results in a polarized M2 phenotype and reduces the production of inflammatory cytokines. Pten is another negative regulator of PI3K signaling, and its absence significantly enhances akt signaling and induces the formation of M2 macrophages [36]. TLR4 is a pathogen-pattern-recognition receptor [37]. TLR4 complex binds to adapter molecules (e.g., LPS) via myeloid differentiation factor 88 (Myd88), thereby enhancing the induction of inflammatory factor expression and inducing macrophages polarization [38]. In addition to the above pathway, it is found that the polarization of TAMs can be induced by the change in a nanocarrier’s surface physical properties [39].

### 2.3. Functions of TAMs

TAMs are an important pro-tumorigenic cell population in the TME, and they play multiple critical roles in tumor development. In this section, we will briefly outline the functions of TAMs in promoting tumor angiogenesis and in affecting metabolism and immunosuppression.

The formation of new blood vessels is essential to provide nutrients and oxygen for further tumor growth. It is known that TAMs are accumulated in hypoxic areas of tumor tissue, and that they secrete various pro-angiogenic molecules including TNF-α, matrix metalloproteinase (MMP), and vascular endothelial growth factor (VEGF) to promote blood vessel formation in tumors [40]. In addition, STAT3 activation in TAMs upregulates the production of angiogenic factors such as VEGF and basic fibroblast growth factor (bFGF), which in turn activates STAT3 signaling in endothelial cells and induces angiogenesis [41]. In addition, TIE2-expressing macrophages are characterized by elevating pro-angiogenic activity and attenuating pro-inflammatory phenotype, which is a receptor for angiostatin and plays an essential function in angiogenesis [42,43]. TAMs promote tumor angiogenesis, cell invasion, and metastasis. During epithelial mesenchymal transition (EMT), tumor cells lose intercellular junctions and apical-basal polarity due to the inhibition of E-cadherin, and consequently acquire the ability to invade [44]. TAMs-derived TGF-β triggers the activation of the β-catenin pathway in the tumor, thereby inducing EMT and accelerating tumor invasion [45]. The extracellular matrix (ECM) provides structural and biochemical support for tumor growth. TAMs provide a variety of ECM regulators, such as MMP and histone proteases, to facilitate tumor cell escape [46]. 

Tumors arise under hypoxic conditions, and tumor cells mainly use aerobic glycolysis to produce energy. TAMs are closely associated with tumor cell metabolism. Studies have reported that TAMs increase glycolysis in NSCLC cells through TNF-α secretion and promote hypoxia through AMP-activated protein kinase and PGC-1α activation, which in turn sustains tumor development [47]. In addition, TAMs have been found to be effective in enhancing aerobic glycolysis and inducing apoptosis resistance in breast cancer cells by transmitting myeloid-specific, HIF-1α-stable, long, non-coding RNA through extracellular vesicles [48]. Furthermore, some secreted molecules of TAMs can shape their immune phenotype. One study showed that ovarian cancer cells promote membrane cholesterol efflux from TAMs. On the one hand, the cholesterol derived from TAMs promotes tumorigenesis, on the other hand, high cholesterol efflux destroys lipid rafts in TAMs, leading to the reprogramming of TAMs to M2 type. The immune response of T-cells is also affected by the metabolites of TAMs. Studies have reported that arginine depletion via Arginase, a marker of M2-like TAMs, inhibits T-cell receptor expression and T-cell cytotoxicity [49].

Tumor immunosuppression is a well-established mechanism for regulating tumor growth. In the early stages of tumorigenesis, M1 macrophages maintain themselves by secreting large amounts of the pro-inflammatory cytokines IFNγ and IL12, which demonstrate anti-tumor activity, and induce the infiltration and activation of cytotoxic T-cells at the tumor site [50]. M1 macrophages can be captured by the tumor, and then transformed to an M2-like state by secreting immunosuppressive cytokines. M2 macrophages inhibit the secretion of IL10 by cytotoxic T cells, while IL10 supports the expression of immunosuppressive regulatory T-cells (Treg) [51]. However, in most types of cancer, TAMs show a higher degree of similarity to anti-inflammatory macrophages and biases toward a pro-tumor state [52]. Numerous studies have shown that TAMs secrete cytokines, such as TGF-β, IL-10, and arginase 1 (Arg 1), which directly inhibit the effector functions of CD4 + and CD8 + T cells, and enhance the expression of Treg cells, thus contributing to the formation of an immunosuppressive microenvironment [53]. TGF-β promotes the polarization of TAMs into M2 phenotypes in the innate immune response, which further promotes TGF-β production and deepens immunosuppression [54]. In the adaptive immune response, TGF-β regulates the production of a variety of cytolytic genes including granzyme A, granzyme B, IFNγ, and FAS ligands, which impair the anti-tumor activity of CD8 + T cells, ultimately leading to pro-tumor TME [55,56]. IL-10 is an important cytokine in the TME, and TAMs-derived IL-10 plays a role in inhibiting IL-12 expression in the autocrine circuit [57]. IL-10 inhibits the release of the cytotoxic cytokine IFN-γ, which is a major stimulus for the differentiation of naïve T-cells, thereby promoting immune evasion [58]. It has been demonstrated that Arg1 metabolizes L-arginine mainly to polyamines and proline, which leads to the dysregulation of T-cell receptor (TCR) signaling, and subsequently induces CD8 + T-cell unresponsiveness [59,60]. Furthermore, a variety of chemokines produced by TAMs have been implicated in immunosuppression. For example, TAM-derived CCL17/CCL22 significantly contributes to the entry of Tregs into the TME via the chemokine receptor CCR4 [61]. CCL18 produced by TAMs attracts naïve T-cells to the tumor site, leading to T-cell unresponsiveness [62].

### 2.4. Therapeutic Strategies Targeting TAMs

**Figure 2:**
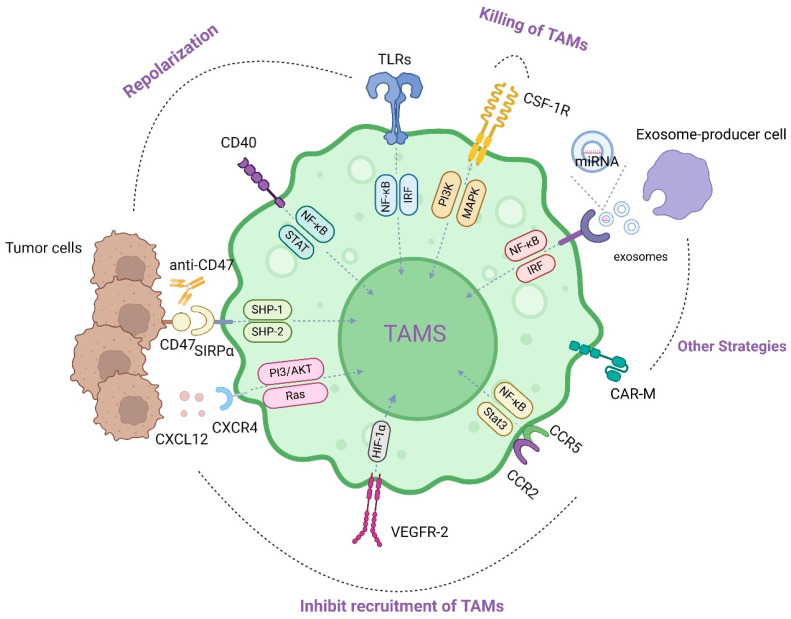
Immunotherapies targeting TAMs. Immunotherapies targeting TAMs have emerged as a potential strategy to enhance anti-tumor immune responses. There are three main categories involved in targeting TAMs: (1) Inhibition of macrophage recruitment: aims to prevent the recruitment of TAMs into the tumor microenvironment. It involves suppressing the production of chemokines like CCL2, CCL5, CXCL12, and their corresponding receptors such as CXCR4. Additionally, inhibition of VEGF formation can also prevent TAMs recruitment by reducing tumor angiogenesis; (2) Killing of TAMs: focuses on directly eliminating TAMs. One way to achieve this is by inhibiting the CSF1-R, which is vital for the survival and proliferation of TAMs. Blocking CSF1-R can lead to the depletion of TAMs within the tumor. The mechanism of action may be related to the activation of the PI3K-dependent pathway and the Ras/mediator-activated protein kinase-dependent pathway; (3) Reprogramming of TAMs into anti-tumor macrophages: aims to convert TAMs from a pro-tumor phenotype into an anti-tumor phenotype. It involves activating specific receptors on TAMs, such as CD40 and TLRs. The mechanism of CD40 can be related to NF-κB and the transcriptional activator STAT. The mechanism of TLR can be related to NF-κB and IRF signaling. Moreover, the binding of CD47 to SIRP-α leads to activation and phosphorylation of the SIRP-α ITIM matrix and recruitment of the SHP-1 and SHP-2 phosphatases, which inhibit the phagocytosis of tumor cells by preventing myosin IIA from aggregating at phagocytic synapses. Interfering with the SIRP-α-CD47 axis has been investigated as a way to restore the recognition and phagocytosis of tumor cells by TAMs. In addition, exosomes and CAR-M have also been studied recently. Exosomes are small vesicles that can be used to deliver therapeutic agents directly to TAMs, allowing targeted modulation of their functions. The mechanism of miRNA can be related to NF-κB and IRF signaling. CAR-M refers to genetically modified macrophages expressing chimeric antigen receptors, enabling them to recognize and eliminate tumor cells more effectively.

Numerous investigations have demonstrated that TAMs represent the most plentiful immune cells infiltrating the TME. TAMs mainly exert M2-like pro-cancer effects in TME, and regulate a variety of malignant effects including angiogenesis, immunosuppression, and metastasis of tumors. Currently, many drugs targeting TAMs have entered the clinical research stage. Therapeutic strategies targeting TAMs include: (1) inhibiting recruitment of TAMs, (2) killing TAMs, (3) regulating the polarization of TAMs [63]. In addition, therapies such as exosomes and CAR-M have also achieved good results in actual treatment. At this stage, drug development for TAMs mainly focuses on the following targets (Figure 2). 

#### 2.4.1. Inhibiting Recruitment of TAMs

A strategy aimed at TAMs is to block their recruitment or prevent monocytes/macrophages penetrating into the tumor. The recruitment of TAMs in tumors is typically driven by monocyte recruitment via the C-C motif chemokine ligand (CCL)-2-C-chemokine receptor (CCR)-2 axis. Alternative pathways engaged in the recruitment of TAMs are C-X-C ligand (CXCL)-12-C-X-C receptor (CXCR)-4 axis and the VEGF receptor pathway. Targeting all of these pathways is effective at inhibiting TAMs recruitment, as described in more detail below. 

Chemokines are small molecular proteins, which perform their function by binding to G protein-coupled chemokine receptors (GPCRs) expressed on the cell surface [64]. The CCL2, also known as monocyte chemotactic protein-1 (MCP-1), was the first CC chemokine discovered and investigated for its inhibition of TAMs recruitment, and it prefers to bind to its receptor, CCR2 [65]. CCL2 is released by tumor cells, stromal cells, and monocytes in TME. Mechanistically, the expression of CCL2 is regulated by the NF-κB signaling pathway. Various stimulatory factors released by tumor cells, such as TNF-α, IL-1β, etc., can activate the NF-κB signaling pathway, thus promoting the expression of CCL2. Inhibition of the NF-κB signaling pathway can reduce the expression of CCL2, thereby reducing the recruitment of TAMs [66]. In addition, it has been reported that STAT3 increases the expression of CCL2, which in turn promotes the infiltration of TAMs [67]. Its receptor, CCR2, has a major part in recruiting bone marrow monocytes into solid tumors, and progression towards TAMs [68]. It has been found that inhibition of the CCL2/CCR2 signaling pathway with anti-CCL2 antibodies can block the recruitment of TAMs, and delay the progression of breast cancer [6]. A clinical trial (NCT01413022) observed that the combination of a CCR2 inhibitor (PF-04136309) with chemotherapy for the treatment of regional pancreatic cancer was safe and well-tolerated—[69]. When compared to chemotherapy on its own, the combination of the CCR2 antagonist CCX827-B (NCT02345408) and the chemotherapeutic agent FOLFIRINOX enhanced overall survival in patients [70]. These studies indicate that blocking the CCL2/CCR2 axis is a potent way to suppress macrophages recruitment. However, it is noteworthy that discontinuation of anti-CCL-2 therapy can lead to rebound, increasing the liberation of monocytes formerly lodged inside the bone marrow, and thus expediting breast cancer metastasis through the promotion of angiogenesis [71]. Therefore, caution should be taken when using anti-CCL2 antibodies for future clinical trials. Apart from CCL2, it is noteworthy that another C motif chemokine ligand, CCL5, also promotes the recruitment of TAMs and contributes to tumor metastasis and recurrence, which can be restricted by the CCL5 receptor antagonist maraviroc and the Raf kinase inhibitory protein [72].

The C-X-C ligand (CXCL)12CXC receptor (CXCR)4 axis and the vascular endothelial growth factor receptor pathway can also inhibit the recruitment of TAMs. Matrix cell-derived CXCL-12 could promote macrophages’ migratory movement across the inner-endothelial barrier and contribute to the aggregation and survival of TAMs in hypoxic areas of the tumor [73]. Targeting CXCR-4 can dramatically lower overall tumor load and metastasis in multiple models of preclinical cancer, including ovarian, prostate, and breast cancers. CXCL12 binding to CXCR4 activates various signaling pathways and promotes cell proliferation and survival. PI3 kinase, Ras, and AKT are all downstream effectors of the CXCL12/CXCR4 axis, and activation of them promotes tumor cell growth, spread, and migration [74,75]. Therefore, inhibiting CXCL-12-CXCR-4 signaling is an active tool for regulating macrophages infiltration and preventing metastasis. A stage I clinical trial (NCT02737072) in advanced refractory solid tumors showed that the combination of the CXCR4 antagonist LY2510924 with durvalumab resulted in a safe and controlled response, with a good overall response of stable condition in four out of nine patients (44.4%) after treatment [76]. VEGF is also involved in the function of recruiting macrophages into the tumor, which requires macrophage-expressed VEGF receptor 2. Huang et al. alleviated the hypoxic environment by using anti-VEGFR2 antibodies. They also reported that low-dose anti-vascular endothelial growth factor receptor 2 inhibited the HIF-1α pathway, improved vascular perfusion, and subsequently repolarized TAMs to the M1 phenotype [77]. Further research found that selective inhibition of VEGF receptor 2 reduces the infiltration of macrophages and reduces angiogenesis in models of breast and pancreatic cancer [78]. 

#### 2.4.2. Killing of TAMs

TAMs exhibit a variety of tumor-promoting effects, and are negatively associated with the prognosis of patients with malignant tumors [79]. Therefore, elimination of existing TAMs is an appealing cancer treatment approach. A strategy has been explored to block colony-stimulating factor 1 (CSF1)-mediated signaling in TAMs. CSF-1, a predominant growth and differentiation factor, widely expresses in cancer cells that cooperate with colony-stimulating factor receptors (CSF-1R) [80]. CSF-1R is a kind of tyrosine kinase receptor expression on monocytes, which could dimerize and transmit signals upon binding to CSF-1 or IL-34 [17]. CSF-1 triggers the dimerization of CSF-1R, and the subsequent autophosphorylation of specific tyrosine residues (e.g., Tyr723) in the intracellular structural domain of CSF-1R [81]. Phosphotyrosine residues in the CSF-1R cytoplasmic domain induce intracellular signaling molecule translocations and activate signaling cascades through SH2 interactions, thereby activating PI3K-dependent and Ras/mitogen-activated protein kinase-dependent pathways [82], ultimately promoting M2-TAMs proliferation, migration, and survival. In addition, blocking the CSF1/CSF-1R axis can reduce the differentiation and recruitment of monocytes to tumor sites, thus further hampering the viability of existing TAMs [83]. Compound D2923 (2-oxo-3,4-dihydropyrimido[4,5-d]pyrimidine derivative) is derived from a series of organic synthesis studies. It is a novel CSF1R-selective inhibitor with potent anti-tumor activity in vitro and in vivo, capable of removing M2-like TAMs from tumors [84]. Apart from that, monoclonal antibodies against CSF1, lacnotuzumab, showed improved patient responses in combination therapy with advanced triple-negative breast cancer in a recent randomized phase II clinical trial (NCT02435680) [85]. Similarly, LY3022855 (NCT01346358) was demonstrated to be useful in reducing levels of TAMs in intractable solid tumors [86]. Besides, a phase 1b/2 study of CSF-1R (ARRY-382), in combination with the PD-1 antibody pembrolizumab for the treatment of individuals with advanced solid tumors (NCT02880371), demonstrated that the combination was effective, with a partial response in 2 patients (10.5%) with pancreatic ductal adenocarcinoma and ovarian carcinoma, who lasted 29.2 months and 3.1 months, respectively. This indicated a great potential of CSF-1R inhibitors in immunotherapy [87].

Apart from that, recent studies have proven that osteoporosis bisphosphonates can remove TAMs by inducing macrophages to produce extraosseous myelin cytotoxicity. In preclinical breast cancer models, bisphosphonates effectively decrease breast tumor growth by reducing the tumor invasion of TAMs [88]. Moreover, it has been found that the anti-tumor drug trabectedin could induce apoptosis of TAMs through receptors of TNF-associated apoptosis-inducing ligand (TRAIL), thereby selectively depleting monocytes or macrophages in blood and tumors [89]. In pancreatic ductal adenocarcinoma, trabectedin effectively depletes monocytes, and changes the TME by reducing IL10 production and inducing CD8 T cell activation, leading to favorable clinical outcomes [90]. Excessive macrophages depletion may disrupt immune homeostasis, as well as increase the incidence of infections and autoimmune diseases. To address the above side effects, we can take measures such as implementing rigorous immune monitoring, promptly detecting immune toxic reactions, and adjusting treatment plans based on the specific situation of the patient. Additionally, combining immunotherapy with other treatment methods, such as chemotherapy or radiotherapy, can better regulate the immune system’s response and reduce immune toxicity; more clinical practice is needed to facilitate the maturation of this therapeutic strategy.

#### 2.4.3. Regulating the Polarization of TAMs

Altering TAMs phenotype is a new potential therapeutic approach to activate anti-tumor immunity. A large number studies have shown that macrophage phenotypes are highly plastic and can be easily regulated by the external microenvironment. This provides an opportunity for TAMs to repolarize into M1-type macrophages, which can effectively fight tumors and activate other immune cells [91]. Recent studies have found that affecting CD40, CD47, TLR, etc., can change macrophages polarization.

CD40 belongs to the TNF receptor superfamily with extensive expression in antigen-presenting cells, including macrophages, dendritic cells, and B cells, through interaction with its ligand CD40 (CD40L). Moreover, the combination of CD40 and CD40L would activate the expression of major histocompatibility complex (MHC) molecules and then release pro-inflammatory cytokines, including IL-12, to promote the activation of T-cells [92]. The most common transcription factors activated by CD40 signaling are NF-κB and activator of transcription STAT [93]. Researchers elucidated that CD40 activation triggered glutamine metabolism and fatty acid oxidation (FAO) to promote ATP citrate lyase-dependent epigenetic reprogramming of pro-inflammatory genes and anti-tumorigenic phenotypes in macrophages [94]. Recently, three cloned CD40 monoclonal antibodies have displayed good anti-tumor effects [95,96,97]: SGN-40, CHIR12.12, and CP-870,893. For example, CP-870,893, a humanized CD40 monoclonal antibody developed by Roche, showed promising efficacy in combination with chemotherapeutic agents, such as paclitaxel. In a phase I clinical trial (NCT00607048), advanced solid tumor patients were treated with CP-870,893 and chemotherapy, resulting in four melanoma patients (27% of patients with melanoma) getting an objective partial response at restaging (day 43) [98]. What is more, Wiehgen et al. found that dual-targeting TAMs blocked by CD40 agonists and CSF-1 transformed TAMs into pro-inflammatory phenotypes [99].

TLR is an innate immune–pattern–recognition receptor that can be activated by invading bacterial particles or viral nucleic acids, leading to macrophages polarization towards the M1 type [100]. TLR agonists (mainly TLR4, TLR7, and TLR9 agonists) are reported to be available for cancer therapy to stimulate polarization of TAMs to a pro-inflammatory phenotype [101]. TLR4 agonists stimulate activator protein 1 and interferon regulatory factor (IRF) 3 signaling, leading to widespread activation of M1-related genes [102]. Studies indicate that cationic polymers for clinical nucleic acid drug therapy can promote therapeutic anti-tumor immunity in a mouse sarcoma model by activating TLR4 signaling to cause the repolarization of TAMs [103]. Currently, TLR7 activation is another attractive target for the repolarization of TAMs as it activates NF-κB and IRF7 signaling in macrophages [102]. TLR7 agonists convert myeloid-derived suppressor cells into tumor-killing M1 macrophages, thereby effectively reversing oxaliplatin resistance in colorectal cancer patients [104]. In addition, TLR9 activation with synthetic unmethylated cytosine-guanine oligodeoxynucleotides (CpG-ODN) leads to the activation of the interleukin 1 receptor-associated kinase 1 (IRAK)/TNF receptor-associated factor (TRAF) pathway, which in turn activates the NF-κB [105]. The TLR9 agonist IMO-2125 can cause anti-tumor macrophages proliferation and tumor regression in mouse models, as assessed in the clinical study of metastatic melanoma [106].

Normal human cells can interact with SIRPα expressed on the surface of macrophages by expressing CD47, and transmit the signal “do not eat me” to macrophages to avoid accidental injury by macrophages [107]. However, tumor cells can evade the killing of macrophages with anti-tumor activity through high expression of CD47. The binding of tumor cell CD47 to macrophage SIRP-α leads to the activation and phosphorylation of the SIRP-α ITIM motif, and recruitment of SHP-1 and SHP-2 phosphatases, which inhibit tumor cell phagocytosis by preventing the accumulation of myosin IIA at phagocytic synapses [108]. Thus, by disrupting with the SIRP-α-CD47 axis (e.g., by using antibodies against SIRPα and CD47), it is possible to restore recognition and phagocytosis of tumor cells by TAMs, and to activate anti-tumor immune responses [107,109]. Anti-CD47 monoclonal antibody Hu5F9-G4 can activate the phagocytosis and other killing effects of macrophages on tumor cells [110]. Clinical trials of its association with rituximab in the treatment of patients with non-Hodgkin’s lymphoma are being conducted in phase 1b/2 (NCT02953509), providing a new approach for tumor immunotherapy [111]. Although blocking CD47-SIRRP alpha is a promising therapeutic strategy, and the monoclonal, double-clonal, and fusion proteins of this target have been extensively studied, off-target toxicity is still a limitation of this strategy.

#### 2.4.4. Other Targeting Strategies

In recent years, treatment strategies for cancer that target TAMs have been rapidly evolving, with numerous relevant research findings. As a new type of drug carrier, exosomes are used for drug delivery as natural nanoscale vesicles with a structure similar to the cell membrane, which can penetrate the biofilm to enter the receptor cell and secrete the drug through biofilm fusion (including endocytosis, cytotoxicity, and phagocytosis). In addition, they have good biocompatibility, targetability, and low immunogenicity [112]. Exosomes do not act directly on tumors, but affect them by releasing internal substances and they contain various substances inside them, such as proteins and miRNAs [113]. MicroRNA, a 19-24 nucleotide non-coding RNA, is a key transcriptional regulator of gene expression in organisms [114]. Mature miRNAs are processed from hairpin-like precursor miRNAs by the RNAse III enzyme double-stranded RNA (dsRNA)-specific endonuclease (DICER) [115]. Deletion of DICER in macrophages induces M1-like TAMs reprogramming, which is characterized by promoting the recruitment of activated CTLs into tumors [116]. MiRNAs are strongly associated with macrophage polarization. Thulin et al. indicated that miRNA-9 in monocytes activated PPARδ, which in turn enhanced M1 macrophages polarization [117]. Furthermore, overexpression of miRNA-125b in macrophages was found to be effective in enhancing macrophage responses to M1-inducing factor IFN-γ by targeting IRF4. Thus, knockdown of IRF4 enhanced M1 activation and pro-inflammatory responses in macrophages [118]. The hypoxic glioma-derived exosome miRNA-1246 promotes glioma proliferation, migration, and invasion in vitro and in vivo by inhibiting the NF-κB signaling pathway, activating the STAT3 signaling pathway and inducing M2 macrophages polarization [119]. The lung cancer exosome miRNA-19b-3p targets PTPRD-mediated dephosphorylation of STAT3 in macrophages, activates STAT3, and causes M2 polarization in macrophages [120]. Exosomes secreted by tumor cells with cell-of-origin-related genetic information can be used as a means of tumor immunotherapy. For example, the presence of miRNA-155 and miRNA-125b in exosomes secreted by pancreatic cancer cells induces a tumor-killing M1 phenotype in macrophages [121]. Similarly, the secretion of miRNA-125b-transfected exosomes from colon cancer mediates macrophage repolarization towards the M1 phenotype [122]. Given the critical role of exosomal miRNAs in macrophage polarization, we believe that exosomes have great potential in clinical therapeutics. Moreover, miRNA vaccines are currently in the developmental stage and offer a promising approach to targeting TAMs and enhancing anti-tumor immunity.

During the past decade, a hot topic of research in immunotherapies has been the chimeric antigen receptor (CAR). It is a single-chain changeable fragment antibody attached to specific proteins in tumor cells. Depending on the intracellular structural domains, CAR has been developed into its fourth generation and has achieved good therapeutic results in hematological tumors. Similar cell therapies such as CAR-M and CAR-NK are inspired by the genetically engineered CAR-T [123]. A central component of CAR-M contains an extracellular structural domain in which a single-chain variable fragment (scFv) offers specific recognition, a hinge structural domain, a membrane-spanning structural domain, and an intracellular structural domain providing outstream signaling [124]. CAR-M expresses pro-inflammatory cytokines and chemokines, promotes the transgenesis from M2 macrophages to M1, recruits antigens and presents them to T cells, and also resists the effects of immunosuppressive cytokines. In addition, it is found that CAR-M is further proven to induce pro-inflammatory TME and to augment anti-tumor T-cell activity in humanized mouse models [125]. CAR-M technology continues to develop, and studies have shown that MPEI/pCAR-IFN-γ could indulge CAR-M1 macrophages to produce potent anti-tumor immunity in vivo. This approach could improve the poor response of CAR-T cell therapy to tumor treatment [126]. A clinical trial (NCT03608618) involved the construction of CAR immune cells (including CAR-M) by transfecting peripheral blood mononuclear cells with mRNA for patients with recurrent/refractory ovarian cancer and peritoneal mesothelioma, but unfortunately the study was discontinued without any results [127]. The CAR family of cellular therapeutics, despite challenges such as off-target toxicity, neurotoxicity, and inflammatory factor storms, also points to a new direction for anti-tumor therapy (Figure 2).

## 3. Clinical Application and Scientific Research in TAMs

Clinical studies targeting TAMs have indeed gained significant attention in the field of tumor therapy. Therapeutic strategies aimed at TAMs include approaches to block their origin and aggregation, as well as to modulate their polarization. These strategies recognize the importance of TAMs in TME, and seek to manipulate their function to enhance anti-tumor immune responses. Clinical research is being conducted to explore novel therapeutic agents and immunotherapies specifically targeting TAMs. Promising results have been observed in liver, breast, lung, and colorectal cancers. This section focuses on clinical studies of TAMs in different types of cancer (Table 1).

### 3.1. Liver Cancer

Hepatocellular carcinoma (HCC) is a highly prevalent malignant tumor of the gastrointestinal system. TAMs are crucial immunological cells present within TME of HCC. It has been demonstrated that TAMs could increase the cell proliferation, invasion, and migration ability of HCC by secreting various cytokines, inducing the occurrence of epithelial–mesenchymal transition, and then accelerating the progression of HCC [128]. In addition, Bao et al. found that DNA stress caused by mitochondrial fission accelerated the infiltration of TAMs in the microenvironment of HCC, and enhanced the growth and metastasis of HCC [129]. Researchers are investigating various approaches to modulate TAMs in HCC to shift their polarization from the immunosuppressive M2 macrophages to pro-inflammatory M1 macrophages, killing or inhibiting the recruitment of TAMs. By doing so, the anti-tumor immune reaction can be enhanced, potentially leading to improved therapy outcomes. Currently, the major drugs for HCC treatment targeting TAMs in clinical or scientific trials are SNDX-6352, BMS-813160, and Sorafenib.

SNDX-6352, a CSFR-1 inhibitor, could affect the proliferation, differentiation, and survival of TAMs by binding to CSF-1R. In a clinical trial (NCT04301778), the combination of SNDX-6352 (CSFR-1 inhibitor) and Durvalumab (MEDI4736) was superior to Durvalumab alone in treating patients with intrahepatic cholangiocarcinoma who had received chemotherapy or radiation therapy. Durvalumab combined with CSF-1R inhibitor SNDX-6352 improved overall survival (OS) in previously treated intrahepatic cholangiocarcinoma patients without causing any serious adverse effects, indicating the great potential of the CSF-1R inhibitor immunotherapy [130].

BMS-813160 is identified as a potent and selective dual CCR2/5 antagonist. Studies have confirmed its ability to inhibit the migration of inflammatory monocytes and macrophages [131]. A clinical study (NCT04123379) of nabulizumab and a CCR2/5 inhibitor (BMS-813160) given before and after surgery to HCC patients is underway, to investigate whether the drug BMS-813160 can boost long-term survival in cancer patients [132].

Sorafenib, a multi-targeted kinase inhibitor that inhibited VEGF2 to reduce the density of TAMs [133], is currently the most effective drug for treating patients with advanced HCC [134]. Numerous preclinical investigations have indicated that sorafenib significantly suppresses tumor growth and lung metastasis [135]. A clinical trial (NCT02971696) to assess the efficacy of sorafenib versus optimal supportive therapy in two cohorts of patients with advanced HCC showed sorafenib (VEGF2 inhibitor) improved overall survival compared with optimal treatment [136]. In addition, a phase II study (NCT01259193) was designed to assess the safety, overall survival, and time to progression of sorafenib in combination with zoledronic acid in advanced HCC. Unfortunately, this clinical trial has been terminated [137].

Recent studies have found that levatinib can regulate cancer immunity in TME by decreasing TAMs, especially exhibiting enhanced anti-tumor activity in the IFN signaling pathway when combined with PD-1 blockers [138]. It is reported that the natural compound baicalin directly induces reprogramming of TAMs into M1-like macrophages via activation of the autophagy-related reticuloendotheliosis virus oncogene homologue B (RelB)/p52. This study demonstrated that the tumor-suppressive effect of baicalin is dependent on the conversion of macrophages for the treatment of HCC from M2-like phenotype to M1-like phenotype [139].

Clinical trials of CAR-M therapies have opened up a new frontier in the use of macrophages to treat solid tumors. An open-label, single-arm, phase I clinical trial (NCT04660929) is underway to assess the tolerability and safety of CT-0508 in terms of the frequency and severity of adverse events in subjects [140]. However, because of the heterogeneity of liver cancer, it remains challenging to discover hepatic tumor cell-specific targets and the effect of engineered macrophages in hepatic TME. Whether CAR-M enhances tumor suppression in combination with CAR-T, multi-targeted kinase inhibitors, and ICIs remains to be further investigated.

### 3.2. Breast Cancer

Breast cancer ranks 2nd in global cancer mortality among females [141]. Abundant laboratory studies have provided evidence of the role of M2-polarized macrophages in breast cancer. These macrophages with an M2-like phenotype have been shown to promote tumor-cell proliferation, interfere with immunosuppression, and promote angiogenesis (the formation of new blood vessels) [12]. The high density of cells expressing macrophage-associated labels in primary breast cancer is usually related to a poorer prognosis for the patient; CD68 and CD163 have been widely used as human pan-macrophage markers [142]. Recently, clinical drugs for TAMs, such as PLX3397, Selicrelumab, Imiquimod, 852A, etc., have been used in clinical trials and have gained good prospects.

Pexidartinib (PLX3397), a CSF1/CSF1R-signaling inhibitor, could induce a reduction in the number of TAMs and lead to a remarkable delay in tumor recurrence [143]. A clinical trial (NCT01596751) assessing the safety and efficacy of eribulin in combination with CSF1 inhibitor PLX3397 in patients with metastatic breast cancer showed that 5 participants in phase II obtained an objective response [144]. Furthermore, a phase 1b study evaluating the safety of PLX3397(NCT01525602) and paclitaxel in advanced solid tumor patients (includes breast cancer) has been completed. The results were promising and without any serious adverse effects in patients receiving PLX3397 1600 mg/day, with 1 in 20 (5%) receiving complete remission, 2 in 20 (10%) obtaining partial remission, and 6 in 20 (30%) with a stable disease [145].

Selicrelumab (also known as RO7009789) is a wholly human IgG2 antibody with strong agonistic activity and low binding affinity for human FcγR. To date, selicrelumab is the most widely evaluated agonist CD40 antibody in clinical research [146]. Apart from being tested as a single therapy, it has been assessed in combination with other therapies. An open-label, multicentre, dose-escalation phase Ib clinical trial (NCT02760797) was designed to evaluate the safety, pharmacokinetics, pharmacodynamics, and therapeutic activity of emactuzumab (CSF-1R) combined with selicrelumab (CD40 Antibody) in patients with advanced solid tumors (including breast cancer) [147]. The results revealed a manageable safety profile and evidence of progressive disease activity of emactuzumab in combination with selicrelumab, but did not translate into objective clinical responses [148].

Imiquimod is an immune response modifier that can induce the immune-mediated rejection of primary cutaneous malignancies when applied locally. It was evaluated in a phase II study (NCT00899574) in patients with cutaneous breast cancer metastases; 10 patients participated and completed the study [149]. The results indicated that patients tolerated the imiquimod treatment well, with only grade 1 to 2 transient topical and systemic adverse effects, consistent with the immunomodulatory effects of imiquimod. Interestingly, the results revealed that topical imiquimod was an active and well-tolerated treatment for skin/chest wall metastases of breast cancer [150].

Molecule 852A, TLR 7 agonist, is a novel immune response modulator. In a phase II trial (NCT00319748), molecule 852A was administered subcutaneously in patients with recurrent ovarian cancer (*n* = 10), breast cancer (*n* = 3), and cervical cancer (*n* = 2) who had been treated with strict therapy [151]. The optimal response was the stabilization of all patients. Unexpected toxicities such as myocardial infarction and infection also occurred [152] (Table 1).

In addition, Li et al. found that Andrographolide suppressed breast cancer progression by regulating TAMs towards M1 polarization via the Wnt/β-catenin pathway [153]. It was found that Cordyceps sinensis extract could inhibit the progression of triple-negative breast cancer by enhancing the polarization of TAMs towards M1-type via activation of the NF-κB pathway [154]. Research found that dendrosomal curcumin exerted a protective effect on metastatic breast cancer in mice by increasing macrophage M1 levels in TME [155]. Immunotherapies targeting TAMs for the treatment of breast cancer have achieved promising results in both clinical research and scientific studies, however there is still a long way to go to bring individualized treatment options to patients.

### 3.3. Lung Cancer

Lung cancer represents one of the most common malignant tumors in the world and its incidence is increasing year by year [141]. Although the treatment of lung cancer has been highly successful, the 5-year survival rate of patients is still less than 15% [156]. Recently, immunotherapy targeting TAMs has received widespread attention. Casanova-Acebes et al. found that early in the course of human and mouse non-small cell lung cancer (NSCLC), tissue-resident macrophages congregated around lung cancer cells and contributed to cancer cell aggression, resulting in an increased number of regulatory T-cells and the promotion of tumor-immune escape. Thus, the removal of tissue-resident macrophages could reduce the quantity of regulatory T-cells, facilitate the accumulation of CD8 + T cells, and inhibit tumor growth [157]. TAMs are critical for the survival of circulating lung cancer cells, and the removal of TAMs by genetic approaches could significantly inhibit cancer cell survival in lung capillaries and lead to lung metastasis [158]. In addition, Lu et al. found that octamer-binding transcription factor 4 (Oct4) expressed by lung cancer cells promoted macrophages’ polarization towards M2-type TAMs through upregulation of M-CSF, leading to tumor growth and the progression of metastasis. These findings indicate that TAMs play indispensable functions in the process of lung cancer proliferation and metastasis, and drugs-targeted TAMs may gain promising results in lung cancer.

Emactuzumab is a CSFR-1inhibitor. It was used in a clinical trial (NCT02323191) combination with PD-L1 (atezolizumab) inhibitor therapy to assess the safety, pharmacokinetics, and activity for participants with advanced solid tumor [159]. The findings indicated that the confirmed objective response rate (ORR) was 12.5% for immune checkpoint blockers (ICB)-experienced NSCLC, and emactuzumab in combination with atezolizumab demonstrated a manageable safety profile, which suggested that the combination of CSFR-1 inhibitors with PD-L1 inhibitors has great promise.

Tazemetostat, is a small molecule enhancer of the zeste homolog 2 (EZH2) inhibitor. It has been found that EZH2 could promote lung cancer metastasis and macrophages infiltration through the up-regulation of CCL5, therefore inhibition of EZH2 could suppress lung cancer progression [160]. An open-label, single-arm, phase Ib/II clinical study (NCT05467748) is currently underway, in which pembrolizumab is being used in conjunction with tazemetostat in the treatment of patients with advanced NSCLC who experienced disease progression from front- or second-line treatment [161].

Eganelisib (IPI-549) is a first-in-class, innovative, oral PI3K-γ inhibitor [162]. Preclinical research revealed that PI3K-γ had an essential role in sustaining the immunosuppressive condition of TAMs in TME. Eganelisib targeting PI3K-γ could reprogram key immunosuppressive macrophages (M2) to anti-tumor macrophages (M1) within TME, thereby down-regulating immunosuppression, increasing immunoreactivity, and eventually leading to the mobilization and multiplication of killer T-cells [163]. A period 1/1b dose-escalation trial (NCT02637531), designed to investigate the safety, tolerability, pharmacokinetics, and pharmacodynamics of IPI-549 single-agent treatment in combination with nivolumab for the treatment of subjects with NSCLC, is in the enrolment phase [164] (Table 1).

Furthermore, scientific studies have found that shuangshen granules could inhibit the growth and formation of lung cancer. The mechanism may be related to the regulation of TAMs [165]. Astragaloside IV blocks macrophage M2 polarization via the AMPK signaling pathway, reducing lung tumor growth, infiltration, migration, and angiogenesis, which might play an active part in inhibiting lung cancer metastasis [166]. Targeting TAMs might be an outstanding strategy for lung cancer treatment, as shown in clinical trials and scientific studies. However, the clinical use of current strategies for treating lung cancer, such as surgery and chemotherapy, is still very limited and we still have a long way to go.

### 3.4. Colorectal Cancer

TAMs play an indispensable role in the pathogenesis of colorectal cancer (CRC). Increasing evidence suggests that TAMs are associated with the prognosis of CRC patients. CD68, as a surface marker of pan-TAMs, is found to be associated positively with overall survival in CRC tissues [167]. From a study of 159 primary CRC, Algars et al. discovered that the count of peritumoral M2 macrophages was positively correlated with survival in earlier-stage CRC, but negatively correlated with survival in stage IV CRC [168]. Furthermore, TAMs in colon cancer secrete TNF-α, IL-1β, and stimulate the NF-κB pathway in the vessel endothelium to generate VEGF, which in turn boosts angiogenesis and alters the TME [169]. Indeed, the findings suggest that TAMs might be a prospective target for new anti-colorectal cancer therapeutic strategies.

Maglumab (Hu5F9-G4), a humanized IgG4 monoclonal antibody, shows high affinity for human CD47. Hu5F9-G4 is designed to disrupt the identification of CD47 by the SIRPα receptor on macrophages, thereby preventing cancer cells from using the “do not eat me” message to avoid macrophage phagocytosis [110]. In preclinical in vivo models, Hu5F9-G4 is active against a variety of solid tumors, including breast, ovarian, colon, liver, brain, and other organ cancers [170,171,172]. A phase 1b/2 clinical trial (NCT02953782) has revealed that 2 in 30 CRC patients confirmed partial remission for 7.0 and 12.5 months, with an objective remission rate of 6.7%, in which Hu5F9-G4 in conjunction with cetuximab were treated for patients suffering from advanced CRC [173]. Moreover, patients with CRC treated with Hu5F9-G4 in combination with cetuximab did not experience any serious adverse effects, and therefore Hu5F9-G4 has a favorable safety and tolerability profile.

Tumor-associated macrophage kinase (TAMK) commonly expresses on tumor cells in the process of malignant transformation. Suppression of TAMK family members AXL and MERTK could lower the threshold of immune activation and enhance anti-tumor immunity. PF-07265807 is a small molecule inhibitor of MERTK and AXL [174]. A clinical trial (NCT04458259) is underway to test the safety, pharmacokinetics, and tolerability of PF-07265807 in participants with advanced CRC, which is still in the recruitment phase [175].

A phase I dose-escalation clinical trial (NCT02777710) was designed to estimate the clinical efficacy and safety of the combination of pexidartinib (anti-CSF1R antibody) and durvalumab (PD-L1 agonist) in individuals diagnosed with advanced/metastatic CRC, and results demonstrated that toxicity was consistent with the expected profile of the individual agents, and no unexpected events were identified with this combination [176] (Table 1).

In addition, scientific studies have found that the nutrient β-carotene is effective at inhibiting M2 macrophages polarization and fibroblast activation, which in turn could suppress the growth and metastasis of CRC [177]. In summary, targeted TAMs therapy has potential efficacy in the cure of CRC, with the deepening of research and continuous technological advancement. It is believed that TAMs therapy will become an important means of CRC treatment. Future clinical studies and clinical applications will further validate its efficacy and safety, bringing more treatment options and hope to CRC patients.

### 3.5. Other Types of Cancer

Other than the cancer types mentioned above, the investigation of TAMs in other kinds of cancer is rapidly evolving, with varying results in patients, and it has attracted widespread attention.

A phase Ib/II trial (NCT02807844) assessed the safety and efficacy of lacnotuzumab (MCS110) versus spartalizumab (PDR001) for patients with pancreatic cancer, endometrial cancer, and advanced melanoma. Common adverse events (AEs) suspected to be drug-related were oedema around the eyes (30%), elevated aspartate aminotransferase (24%), and elevated blood creatine phosphokinase (24%), with the most common grade ≥3 AEs suspected to be related to the drug (6%). Out of 30 patients suffering from pancreatic cancer, 1 achieved a partial response (346 days into the study) and 2 had durable stable disease (328 and 319 days into the study, respectively). The results showed that MCS110, combined with PDR001, was generally well tolerated [178].

In addition, a clinical trial (NCT01676831) found a favorable safety profile with the topical use of raquinimod (TLR7/8 agonist) in treating early-stage cutaneous T-cell lymphoma [179]. However, due to the small number of subjects, further in-depth studies are needed. What is more, a clinical trial [180] (NCT00537368) evaluated the safety, efficacy, and pharmacokinetics of CNTO888(CCL2 antibody) alone or in combination with other commonly used chemotherapeutic agents for the therapy of solid tumors such as ovarian and prostate cancers. Durable disease stabilization of 5–15.7 months was observed in 4 patients. Moreover, two of these patients with ovarian and prostate cancer experienced sustained reductions in cancer antigen 125 and prostate-specific antigen of more than 50% at 10 and 5 months, respectively [181]. In addition, a clinical study (NCT00992186) designed to determine the efficacy and safety of carlumab in patients with metastatic refractory prostate cancer demonstrated that carlumab was well tolerated but failed to show single-agent anti-tumor activity [182]. For individuals with a recurrent or refractory primary malignant central nervous system tumor or freshly diagnosed diffuse endogenous pontine gliomas, a phase I clinical trial (NCT03389802) is currently underway, assessing the site-specific tolerability and safety of a monoclonal antibody to CD40 agonist (APX005M) in patients [183]. Furthermore, histone deacetylase inhibitors were found to be effective in modulating the polarization of TAMs towards the M1-type, and thus exerting anti-tumor effects [184]. A phase II clinical trial (NCT00134043) was conducted to estimate the objective response to a histone deacetylase inhibitor (vorinostat) in 19 participants with progressive thyroid cancer. The results showed that there were no patients achieved partial or complete remission [185] (Table 1).

Clinical studies and scientific research are helping us to gain a deeper understanding of the relationship between TAMs and different types of cancer. These studies are expected to provide an important theoretical basis for the exploitation of new drugs and immunotherapeutic approaches. In the future, more personalized and effective treatment options can be provided to patients.

**Table 1:** Relevant clinical trials of immunotherapies targeting TAMs in different types of cancer.

Clinical Trials.gov Identifier	Target	Drug	Study Design	Type	Patients	Outcomes	Status	References
NCT01413022	CCR2	PF-04136309	Oxaliplatin + Irinotecan + Leucovorin + Fluorouracil vs. Oxaliplatin + Irinotecan + Leucovorin + Fluorouracil + PF-04136309	Pancreatic Neoplasms	44	TCR↑	Completed	[69]
NCT02345408	CR2	CCX872-B	CCX872-B	Pancreatic Cancer	54	PFS↑	Completed	[70]
NCT02737072	CXCL-12-CXCR-4	LY2510924	LY2510924 + Durvalumab	Solid Tumor	9	Safe	Completed	[76]
NCT02435680	CSF1	Lacnotuzumab	Lacnotuzumab + Larboplatin + Gemcitabine vs.Carboplatin + Gemcitabine	Advanced Triple Negative Breast Cancer	50	PFS↑	Completed	[85]
NCT01346358	CSF-1R	LY3022855	LY3022855	Neoplasms	72	Well Tolerated	Terminated Has Results	[86]
NCT02880371	CSF-1R	ARRY-382	ARRY-382 + Pembrolizumab	Advanced Solid Tumors	82	ORR↑	Completed	[87]
NCT00607048	CD40	CP-870,893	Paclitaxel + Carboplatin + CP-870,893	Solid Tumor	34	Safe	Completed	[98]
NCT02953509	CD47	Magrolimab	Magrolimab + Rituximab vs.Magrolimab + Rituximab + Gemcitabine + Oxaliplatin	Non Hodgkin Lymphoma	178	ongoing	Active, not recruiting	[111]
NCT03608618	CAR-M	MCY-M11	MCY-M11 + Cyclophosphamide	Advanced Ovarian Cancer and Peritoneal Mesothelioma	14	unknow	Terminated	[127]
NCT04301778	CSF-1R	SNDX-6352	Durvalumab vs. SNDX-6352	Intrahepatic Cholangiocarcinoma	5	OS↑	Completed	[130]
NCT04123379	CCR2	BMS-813160	Nivolumab vs.BMS-813160	Hepatocellular Carcinoma	36	Ongoing	Active, not recruiting	[132]
NCT02971696	VEGF2	Sorafenib	Sorafenib vs. Best Supportive Care	Hepatocellular Carcinoma	55	OS↑	complete	[136]
NCT01259193	VEGF2	Sorafenib + Zoledronic Acid	Sorafenib + Zoledronic Acid	Hepatocellular Carcinoma	50	Unknow	Terminated	[137]
NCT04660929	CAR-M	CT-0508	CT-0508 vs.CT-0508 + Pembrolizumab	Hepatocellular Carcinoma	48	Ongoing	Recruiting	[140]
NCT01596751	CSF1	PLX3397	PLX3397 vs. Eribulin	Metastatic Breast Cancer	67	PFS↑	Completed	[144]
NCT01525602	CSF1	PLX3397	PLX3397 + Paclitaxel	Breast Cancer	74	Relief Degree↑	Completed	[145]
NCT02760797	CD40	RO7009789	Emactuzumab + RO7009789	Metastatic Triple-Negative Breast Cancer	38	PFS↑	Completed	[147]
NCT00899574	TLR7	Imiquimod	Imiquimod	Breast Cancer	10	Relief Degree↑	Completed	[149]
NCT00319748	TLR7	852A	852A	Breast Cancer	15	Relief Degree↑	Completed	[151]
NCT02323191	CSF-1R	Emactuzumab	Atezolizumab vs. Emactuzumab	Lung Cancer	221	Relief Degree↑	Completed	[159]
NCT05467748	CCL5	Tazemetostat	Tazemetostat	Nonsmall Cell Lung Cancer	66	Ongoing	Not yet recruiting	[161]
NCT02637531	PI3K-γ	IPI-549	IPI-549 vs.IPI-549 + Nivolumab	Nonsmall Cell Lung Cancer	219	Ongoing	Active, not recruiting	[164]
NCT02953782	CD47	Hu5F9-G4	Hu5F9-G4 + Cetuximab	Colorectal Cancer	78	ORR↑	Completed	[173]
NCT04458259	TAMK	PF-07265807	PF-07265807 vs. PF-07265807 + Sasanlimab vs. PF-07265807 + Sasanlimab + Axitinib	Neoplasm Metastasis	67	Ongoing	Active, not recruiting	[175]
NCT02777710	CSF-1R	Pexidartinib	Pexidartinib + Durvalumab	Colorectal Cancer	48	ORR↑	Completed	[176]
NCT02807844	CSF-1R	MCS110	MCS110 vs.MCS110 + PDR001	Pancreatic Carcinoma Melanoma Endometrial Carcinoma	141	Safe, Well-tolerated	Completed	[178]
NCT01676831	TLR7/8	Resiquimod	Resiquimod	Cutaneous T Cell Lymphoma	13	Safe	Completed	[179]
NCT00537368	CCL2	CNTO 888	CNTO 888	Cancer	44	Safe	Completed	[180]
NCT00992186	CCL2	Carlumab	Carlumab	Prostate Cancer	46	OS↑PFS↑	Completed	[182]
NCT03389802	CD40	APX005M	APX005M	Central Nervous System Tumor	32	Ongoing	Recruiting	[183]
NCT00134043	Histone Deacetylase Inhibitor	Vorinostat	Vorinostat	Thyroid Cancer	19	No Relief	Completed	[185]
NCT02371369	CSF-1R	Pexidartinib	Pexidartinib vs. Placebo	Pigmented Villonodular Synovitis Giant Cell Tumors of the Tendon Sheath Tenosynovial Giant Cell Tumor	120	CR↑ PR↑	Completed	[186]
NCT01444404	CSF-1R	AMG 820	AMG 820	Advanced Solid Tumors	25	Safe	Completed	[187]

Abbreviations: tumor control rate (TCR), progression-free survival (PFS), overall survival (OS), objective response rate (ORR), partial response (PR), and complete response (CR).

## 4. Conclusions and Future Directions

TAMs represent a major category of innate immune cells in TME, and they are broadly expressed in a diverse range of tumor tissues [188]. A large infiltration of TAMs or the abundance of TAM-related genes generally predicts tumor advancement or a worse outcome of the disease. There are two main forms of TAM: M1-type macrophages with tumor growth suppression, and the M2-type macrophages with a tumor-promoting effect, which can be transformed through multiple signaling pathways. In addition, extensive findings have indicated that TAMs are closely related to various tumor-related processes, promoting oncogenesis and proliferation, speeding up angiogenesis, facilitating invasion and metastasis, and triggering drug tolerance and immunosuppression. Therefore, multiple tumor-promoting mechanisms in TAMs offer many attractive new targets in cancer therapy. Tumor immunotherapies that target TAMs, involving the suppression of TAMs recruitment, the acceleration of TAMs apoptosis, and the modulation of TAMs anti-tumor polarity, display tremendous potential in both basic and clinical oncology studies.

It is inspiring that drugs against TAMs are undergoing clinical trials and displaying anti-tumor benefits. The CSF-1R inhibitor pexidartinib shrank tumor size and improved symptoms in patients with tenosynovial giant cell tumor (TGCT), with encouraging results in the phase III clinical study (NCT02371369) of CSF1/CSF1R-targeted therapy for benign diffuse TGCT [186]. Results from the ENLIVEN trial prompted the FDA to approve pexidartinib for the treatment of asymptomatic TGCT patients with serious comorbidities or functional limitations TGCT, which originated in the synovium and was marked by CSF-1 overexpression, and this supported a successful response to treatment [189]. However, some poor therapeutic outcomes also exist. In a phase I/II trial of AMG 820-pembrolizumab co-therapy, 37.1% had rash and maculopapular rash, 48.3% had periorbital and facial oedema, and 59.5% of patients had elevated aminotransferase of aspartic acid [190]. Furthermore, in a phase I trial [187] of AMG 820 monotherapy (NCT01444404), periorbital oedema was present in 44% of participants and aspartate aminotransferase was elevated in 28%. It has been argued that the limited success of the current published data may be attributed to acquired disease resistance mediated through the activation of phosphatidylinositol-3-kinase [191]. Taken together, these data indicate that targeting macrophage subpopulations rather than all macrophage populations may have a prospective therapeutic effect. Therapeutic strategies targeting CCL2-CCR2 signaling to kill TAMs have proved promising in existing studies, but there are some problems, e.g., monocytes require CCL2-CCR2 signaling to enter the bloodstream from the skeleton, the inhibition of CCL2 leads to the severe depletion of monocytes, and macrophages in the tissues may undergo compensatory proliferation if TAMs recruitment is blocked. In fact, the discovery of molecules required to inhibit monocyte retention (e.g., CCL3) or to induce monocyte differentiation may be a superior approach [192]. Taken together, these observations suggest that repolarization of macrophages is the most promising therapeutic strategy available. It is well known that TAMs are typically tumor-friendly, however, they can be converted to a tumor cell-killing state under certain conditions, and repolarization can rebalance microenvironmental immunity from a therapeutic pro-tumor immune infiltration to a state of active tumor-rejecting immune infiltration. Furthermore, this approach does not have the drawbacks caused by the inhibition of TAMs recruitment and the long-term toxicity associated with a lack of TAMs. Currently, imiquimod (TLR7 agonist) is permitted by the FDA for topical application in squamous cell carcinoma and basal cell carcinoma. Furthermore, the emerging therapeutic technology CAR-M has created a new paradigm for the exploitation of macrophage-based cancer immunopharmaceuticals. On 27 July 2020, the US Food and Drug Administration granted new drug approval for anti-human HER2-CARM (CT-0508) for the treatment of recurrent or metastatic HER2 overexpressing solid tumors. It marks a meaningful advance in cell-based cancer immunotherapy.

As far as I am concerned, for different cancers, targeted therapy with TAMs in HCC is promising because TAMs are known to infiltrate to a high degree in HCC, which correlates with the prognosis of the tumor. Secondly, recent studies have found that xanthine oxidoreductase (XOR) deficiency in TAMs increased isocitrate dehydrogenase (IDH)-3α activity, which polarized TAMs towards alternatively activated M2 phenotypes, exacerbated CD8 + T cell depletion, and promoted HCC progression. Among them, XOR was usually considered to be a promoter of M1 macrophages activation, therefore, XOR-IDH3α was a key axis controlling TAMs polarization and HCC progression [193]. The discovery of this potential target provides novel insights into the treatment of HCC. Moreover, recent findings have linked a number of genetic mutations in thyroid cancer that are associated with the polarization of TAMs, for instance, mutations in the *BRAF* gene have been shown to result in the conversion of TAMs to the M2 phenotype [194], and targeted therapies can be much better exploited with the benefit of this gene. Immunotherapies targeting TAMs have different immunotherapeutic effects in different tumors due to the heterogeneity of tumors and differences in the individual immune microenvironment of patients. TAMs immunotherapies also do not show good therapeutic efficacy in all individuals and on all tumors, and the occurrence of adverse events is not the same.

In conclusion, TAMs hold high promise as targets for cancer immunotherapy. Currently, targeted therapeutic drugs for TAMs are still in the research and development stage, and the targeting and selectivity of the drugs need to be further improved, while the diversity and plasticity of TAMs in TME also increase the challenge of drug therapy. TAMs play an immunosuppressive part in tumors, and can inhibit the killing function of immune cells; M2 TAMs are especially key to tumor refractoriness and the development of drug resistance. Therefore, overcoming tumor immune escape is crucial for therapies targeting TAMs. Tumor cells and TAMs can acquire drug resistance through multiple mechanisms, which poses a challenge for therapies targeting TAMs. More effective drugs and combination therapy strategies need to be developed to overcome resistance. Going forward, better strategies would be to specifically target tumor-promoting macrophages, enhance the anti-tumor activity of macrophages, or depolarize existing macrophages. Currently among the strategies targeting TAMs complexes, exosomes and CAR-M therapies show potential for treating solid tumors. Although some therapeutic strategies targeting TAMs have displayed some effectiveness in the data from clinical trials, there is a dearth of adequate clinical data and evidence to support their widespread use. Further large-scale clinical studies are needed to address the safety and tolerability issues associated with the treatment, with the ultimate goal of optimizing treatment options for patients.

## Data Availability

Not applicable.
